# Assessing the Diagnostic Accuracy of Physicians for Home Death Certification in Shanghai: Application of SmartVA

**DOI:** 10.3389/fpubh.2022.842880

**Published:** 2022-06-17

**Authors:** Lei Chen, Tian Xia, Rasika Rampatige, Hang Li, Tim Adair, Rohina Joshi, Zhen Gu, Huiting Yu, Bo Fang, Deirdre McLaughlin, Alan D. Lopez, Chunfang Wang, Zheng'an Yuan

**Affiliations:** ^1^Shanghai Municipal Center for Disease Control and Prevention, Shanghai, China; ^2^Shanghai Institutes of Preventive Medicine, Shanghai, China; ^3^Melbourne School of Population and Global Health, The University of Melbourne, Melbourne, VIC, Australia; ^4^Faculty of Medicine, The George Institute for Global Health, University of New South Wales, Sydney, NSW, Australia; ^5^The George Institute for Global Health, New Delhi, India; ^6^Vital Strategies, New York, NY, United States; ^7^Department of Health Metrics Sciences, IHME, University of Washington, Seattle, WA, United States

**Keywords:** Smart Verbal Autopsy, cause of death, CRVS system, demography, epidemiology

## Abstract

Approximately 30% of deaths in Shanghai either occur at home or are not medically attended. The recorded cause of death (COD) in these cases may not be reliable. We applied the Smart Verbal Autopsy (VA) tool to assign the COD for a representative sample of home deaths certified by 16 community health centers (CHCs) from three districts in Shanghai, from December 2017 to June 2018. The results were compared with diagnoses from routine practice to ascertain the added value of using SmartVA. Overall, cause-specific mortality fraction (CSMF) accuracy improved from 0.93 (93%) to 0.96 after the application of SmartVA. A comparison with a “gold standard (GS)” diagnoses obtained from a parallel medical record review investigation found that 86.3% of the initial diagnoses made by the CHCs were assigned the correct COD, increasing to 90.5% after the application of SmartVA. We conclude that routine application of SmartVA is not indicated for general use in CHCs, although the tool did improve diagnostic accuracy for residual causes, such as other or ill-defined cancers and non-communicable diseases.

## Introduction

Accurate data on causes of death are essential for policymakers and public health experts to plan appropriate health policies and interventions to improve population health. In Shanghai, a mega-city with a population of 24 million, the vital statistics registration system registers almost all deaths of the resident (Hukou) population (1). Deaths that occur in the hospital are certified by the attending doctor. For the 30% of deaths in Shanghai that occur at home or are otherwise not medically attended, the family members of the deceased present to Community Health Centers (CHC), usually with available medical documentation, such as discharge summaries, medical records, and laboratory test results, and the CHC doctor on duty reviews the records and issues a death certificate. In such cases, the recorded cause of death (COD) may be less reliable than that for hospital death.

Verbal Autopsy (VA) is a practical method that can help determine causes of death in regions where most deaths occur at home or where medical certification is limited or unreliable (2, 3). Automated VA does not require physician review of the responses to a questionnaire to ascertain signs and symptoms preceding death; rather, the most probable COD is predicted from the application of a diagnostic algorithm. Where physicians are available to immediately review the outputs of a verbal autopsy and certify the COD, a specific tool, SmartVA for Physicians, has been developed to facilitate physician diagnoses. This innovation produces a summary of all endorsed symptoms, as reported by family members, providing more information for the certifying physician to determine the COD for people who die outside of hospitals (4, 5). The validity of Smart VA as a diagnostic tool has been demonstrated in a diverse range of low- and middle-income populations (6–10).

To ascertain whether routine application of the method would improve the quality (i.e., diagnostic accuracy) of death certification in Shanghai (especially for deaths occurring outside health facilities), SmartVA for Physicians was applied to a sample of community deaths for which the true cause had been separately established *via* an independent medical record review study. The findings were compared with diagnoses from routine practice to ascertain the value, if any, of incorporating Smart VA into the diagnostic practices of physicians in Shanghai certifying the cause of home deaths.

## Materials and Methods

### SmartVA Auto-Analyze Package

The SmartVA Auto-Analyze is a software package that builds on SmartVA Analyze and includes the Population Health Metrics Research Consortium (PHMRC) shortened VA questionnaire, the Open Data Kit (ODK) suite for data collection, and the modified Tariff 2.0 algorithm for computer analysis of the VA interview responses (11–13). The SmartVA Auto-Analyze was developed to be used by physicians in real time, and produces a list of up to three most likely causes of death at the individual level, commonly referred to as SmartVA for Physicians (for brevity, we use the term SmartVA in this article). The PHMRC shortened questionnaire was validated in terms of quantifying the decline in diagnostic accuracy as a function of deleting symptom questions in the long form of the questionnaire, using formal item reduction methods (14). Subsequently, the shortened questionnaire has been applied to selected China CDC sites and validated against local diagnostic practices in these sites (15).

### Training and Administration

A local VA team, trained by experts in SmartVA from the University of Melbourne, trained 32 CHC doctors as VA interviewers. User manuals with detailed instructions and Standard Operating Procedures (SOP) were introduced during the training and were made available for use by the Shanghai Municipal Center for Disease Control and Prevention (SCDC) project staff. In addition, the interviewers received training on correct death certification practices as well as training on operating Android-based tablets to conduct SmartVA interviews and implement troubleshooting. After the training, the interviewers underwent supervised field practice to ensure that they had the requisite skills and conceptual knowledge to carry out VAs as required.

A local information technology (IT) technical/data management staff member, with support from the University of Melbourne technical team, installed the Open Data Kit Collect software, the electronic SmartVA questionnaire and media file onto tablets, and SmartVA-Auto-Analyze onto computers, and prepared all devices for SmartVA data collection.

### Data Collection and Diagnostic Procedures

Previous experience with similar validation studies suggests that at least 20 gold standard (GS) cases are required for each cause to establish the COD accuracy and validity within acceptable uncertainty bounds (4). For investigating diagnostic accuracy of the top 20 causes of death, therefore, at least 400 GS cases were required. To allow for VA interview refusals, poor quality medical records to establish GSs, etc., we applied multistage sampling to select 16 community health centers (CHC) from three districts, chosen as representative of urban, suburban, and urban-suburban areas in Shanghai. Minhang district, Songjiang District, and Pudong District, each of which contains urban, suburban, and urban-suburban areas were first selected. Then, five CHCs from Minhang district, five CHCs from Songjiang district, and six CHCs from Pudong district were selected to meet our stratification criteria. All home deaths (1,648) in these CHCs which met our inclusion criteria were eligible for inclusion in our study, although it was expected that the final number of cases would be lower due to refusals, medical record quality and availability, etc.

Each home death that occurred between December 2017 and June 2018 was investigated by a trained CHC doctor on duty. Doctors identified an appropriate respondent (>18 years of age, cared for the deceased, or most familiar with the symptoms and terminal phase of the deceased) from among the family members who came to report the death to the CHC, requested their consent to participate in the pilot study, and interviewed them.

**Figure 1:**
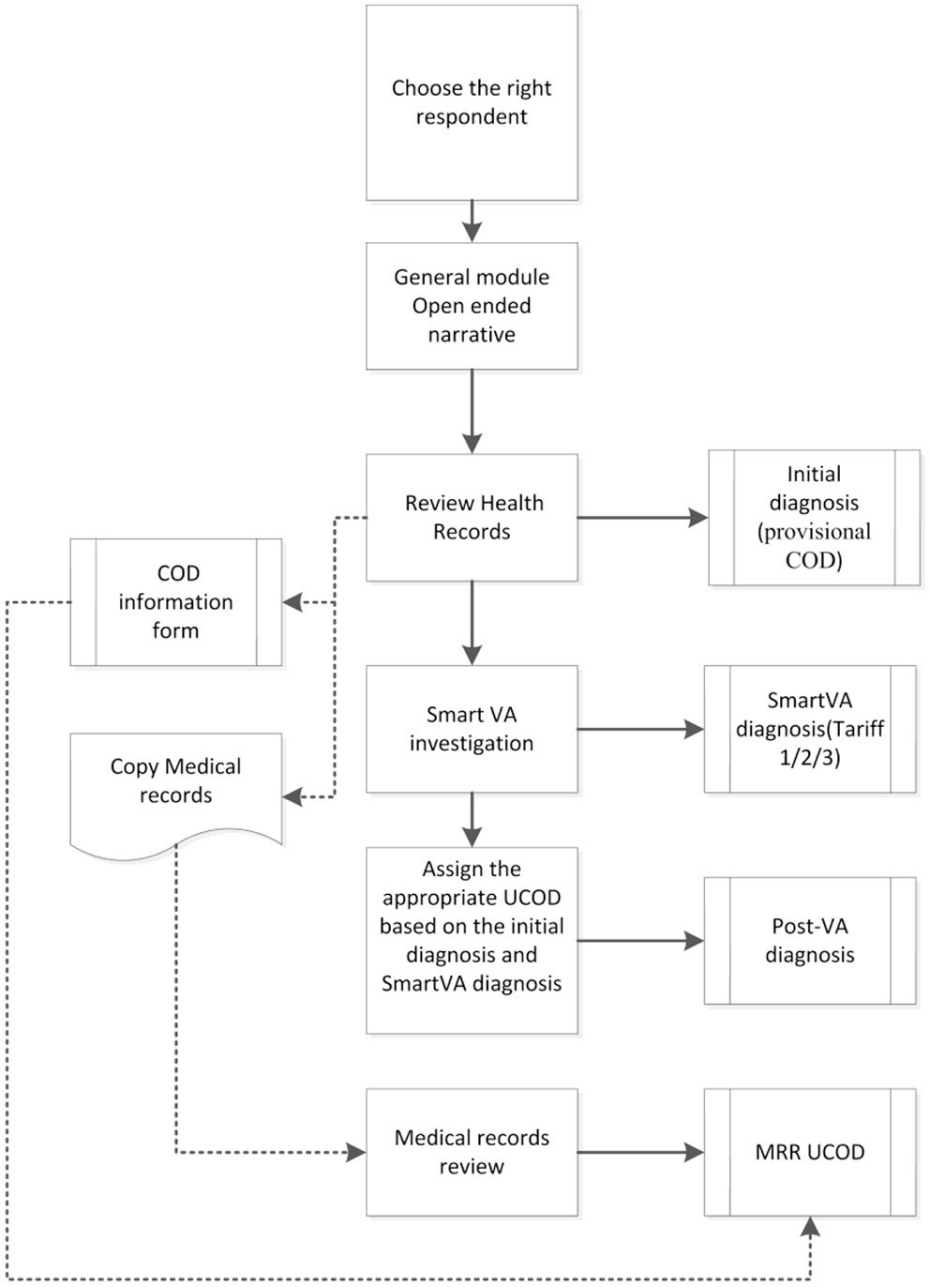
The field implementation procedures for this study.

The various diagnoses associated with each case included in the study are shown in Figure 1. At the end of the interview, the CHC doctor assigned an **Initial diagnosis** with an underlying cause of death (UCOD) selected according to usual practice and procedures in place, which included a review of the outpatient clinical records and any other documentation brought by the family when reporting the death to the CHC. Next, the physician ran the SmartVA-Auto-Analyze program for each death which suggested up to three possible UCOD; these predicted diagnoses from the Tariff diagnostic algorithm are labeled as the **SmartVA diagnosis (Tariff COD 123)**. Finally, the physician then reviewed the **Initial diagnosis** in the context of the additional information provided by the SmartVA diagnoses, including the list of endorsed symptoms provided by SmartVA, and used this information to assign a **Post-VA diagnosis (as shown in** Figure 1).

### Ethics Approval

Ethics approval was obtained from Shanghai CDC (Ethics ID: 2016-28) and the University of Melbourne Ethics Committees (Ethics ID: 1647517.1.1). All participants were provided with a participant information sheet and consent forms in the local language.

### Monitoring and Evaluation

Each CHC doctor was asked to complete a Microsoft Excel spreadsheet (“COD information form” box in Figure 1) with the data on demographics, initial diagnosis, SmartVA (Tariff) diagnosis, and the post-VA diagnosis of the UCOD for each case. This spreadsheet was submitted to SCDC by the CHC doctor at the end of each month, for monitoring the progress and quality of the study implementation. After 6 months of data collection, a program manager from SCDC integrated the data from all 16 CHCs and performed further analysis.

### GS UCOD and Data Analysis

The medical records for all deaths for which a VA was carried out in these three districts were carefully evaluated by an independent Medical Record Review (MRR) team. The medical records of each home deaths were carefully audited according to the *ex-ante* study protocols adopted from the PHMRC study The MRR team members were experienced district CDC coders/physicians. The members were trained on how to review a medical record by the University of Melbourne team, as well as in the definition and interpretation of the standard diagnostic criteria and GS levels. The MRR team assigned each death a “GS” UCOD, which we define here as the **MRR UCOD**, based on the GS criteria for each COD developed by the PHMRC, and as applied in several studies (4, 16–18). Under these criteria, GS1 refers to the highest standard of (i.e., confidence in) diagnostic accuracy of the UCOD, progressing down to GS4, for which diagnostic confidence following the MRR was lowest. For example, the GS1 criteria for a case to be diagnosed as lung cancer is based on histological confirmation, whereas GS4 would be used for cases where the MRR concluded that there was unsupported clinical diagnosis.

Causes of death from the application of SmartVA, as well as the UCOD from the MRR, were transformed to the SmartVA cause list (as shown in Supplementary File 1) to facilitate comparison, given this was an abbreviated list of causes as appropriate for VA. Based on the Smart VA cause list, we carried out the following comparisons: (i) concordance between the initial diagnosis and MRR UCOD (to ascertain the accuracy of current diagnostic practice); and (ii) concordance between the initial and post-VA diagnosis (to ascertain the impact of applying SmartVA on diagnostic accuracy). In addition, we developed a misclassification matrix by cause to identify the pattern and extent of certification errors. For the misclassification matrices, only the 16 leading causes of death based on MRR UCODs have been included to facilitate interpretation of findings; all other diseases were merged into a residual group, labeled “others.”

Standard validation metrics, such as sensitivity, positive predictive value (PPV), Cohen's kappa, chance-corrected concordance (CCC), and cause-specific mortality fraction (CSMF) accuracy, were calculated to assess concordance. The statistical analysis was performed using R 3.6 software.

## Results

Of the 1,648 deaths reported to the study CHCs during the defined period, only 619 (37.6%) could be included in this study. This was because many cases did not meet the study's inclusion criteria for eligible respondents or refused to participate. Of the 619 deaths for which a SmartVA interview was conducted, 570 cases also had available medical records that enabled the establishment of a GS1 and GS2 diagnosis following MRR.

There was no significant difference in the age and sex composition between the 570 deaths and the total number of CHC deaths in same area and time period (as shown in Table 1; *p* = 0.862 for sex and *p* = 0.135 for age). The majority of deaths were among those aged 70 years and above.

**Table 1:** Age-sex distribution of home deaths and deaths investigated by Smart Verbal Autopsy (SmartVA) in the 16 community health centers (CHCs).

**Age**	**Total deaths reported by CHCs**				**Smart VA cases**			
	**Male (%)**	**Female (%)**	**Total**	**Male (%)**	**Female (%)**	**Total**		
			***N***	**%**			***N***	**%**
<49	4.2	1.3	46	2.8	2.1	2.1	12	2.1
50–69	20.5	10.1	253	15.3	23.9	13.3	106	18.6
70~	75.3	88.5	1,349	81.9	73.9	84.6	452	79.3
Total (%)	50.4	49.6			49.8	50.2		
Total (*N*)	830	818	1,648		284	286	570	

The CSMFs for all the home deaths in the 16 CHCs in 2017, and the CSMFs based on the VA results from this study, conducted in the same 16 regions in 2018, showed a similar COD distribution based on the common SmartVA cause list (as shown in Table 2).

**Table 2:** The cause-specific mortality fractions (CSMFs) of home deaths in the 16 regions of Shanghai in 2017 and in 2018.

**Rank**	**Home deaths in 2017 (before VA)**		**Home deaths in 2018 (Smart VA)**	
	**Leading cause of death**	**(%)**	**Leading cause of death**	**(%)**
1	Stroke	19.2	Stroke	17.8
2	Ischaemic heart diseases	15.2	Other cancers	15.6
3	Chronic respiratory diseases	12.3	Ischaemic heart diseases	12.6
4	Other cancers	11.7	Lung cancer	12.1
5	Lung cancer	7.8	Chronic respiratory diseases	11.2
6	Other non-communicable diseases	6.4	Stomach Cancer	4.9
7	Diabetes	4.3	Other non-communicable diseases	4.5
8	Undetermined	4.2	Colorectal cancer	3.3
9	Stomach cancer	3.6	Other cardiovascular diseases	3.0
10	Colorectal cancer	2.7	Falls	2.8
11	Falls	2.7	Diabetes	2.4
12	Other cardiovascular diseases	1.9	Esophageal cancer	1.7
13	Esophageal cancer	1.2	Leukemia/lymphoma	1.7
14	Leukemia/lymphoma	1.2	Other Infectious Diseases	1.4
15	Prostate cancer	1.1	Other injuries	1.4

From the MRR of the deaths analyzed by SmartVA for Physicians, stroke was the leading COD, accounting for 17.8% of deaths, followed by other cancers (15.6%) and ischemic heart disease, lung cancer, and chronic respiratory diseases (CRDs), accounting for 12.6, 12.1, and 11.2% of deaths, respectively. All other causes accounted for <5% of deaths. Broadly speaking, CSMFs for causes of death diagnosed by SmartVA were similar to those based on existing diagnostic practices in Shanghai, with only slight changes in the ranking of causes of death (Table 2).

The concordance between the initial diagnosis and the MRR UCOD (assessing the accuracy of existing diagnostic practices) and between the post-VA diagnosis and MRR UCOD (assessing the impact of SmartVA on diagnostic accuracy) was measured using chance-corrected concordance (CCC; Tables 3–5). This metric evaluates the extent of agreement (average sensitivity) of individual diagnoses between the two sources, corrected for chance. Additionally, the CSMF accuracy was evaluated by measuring the absolute deviation of the CSMFs for the initial diagnosis of the SmartVA CSFMs from the MRR UCOD (12, 19, 20). The closer this value is to 1, the higher the concordance of the results.

**Table 3:** Validation metrics comparing initial diagnosis or post-VA diagnosis with Medical Record Review (MRR) underlying cause of death (UCOD) (top 15 specific UCOD).

**Rank**	**UCOD**	**Initial diagnosis**						**Post-VA diagnosis**					
		**Sensitivity**	**PPV**	**Kappa**	**CCC**	**CSMF**	**CSMF** **accuracy**	**Sensitivity**	**PPV**	**Kappa**	**CCC**	**CSMF**	**CSMF** **accuracy**
1	Stroke	0.94	0.87	0.88	0.94	19.30		0.96	0.88	0.90	0.96	19.50	
2	Other cancers	0.88	0.96	0.90	0.87	14.20		0.94	0.93	0.93	0.94	15.80	
3	Ischaemic heart diseases	0.89	0.85	0.85	0.88	13.20		0.93	0.89	0.90	0.93	13.20	
4	Lung cancer	0.97	0.99	0.98	0.97	11.90		0.97	1.00	0.98	0.97	11.80	
5	Chronic respiratory diseases	0.83	0.93	0.86	0.82	10.00		0.91	0.95	0.92	0.90	10.70	
6	Stomach cancer	0.96	0.90	0.93	0.96	5.30		1.00	0.93	0.96	1.00	5.30	
7	Other non-communicable diseases	0.58	0.83	0.67	0.55	3.20		0.81	0.78	0.78	0.79	4.70	
8	Colorectal cancer	0.95	0.86	0.90	0.94	3.70		1.00	0.95	0.97	1.00	3.50	
9	Other cardiovascular diseases	0.29	0.71	0.41	0.25	1.20		0.24	0.67	0.34	0.18	1.10	
10	Falls	0.81	0.81	0.81	0.80	2.80		0.69	0.85	0.75	0.67	2.30	
11	Diabetes	0.71	0.67	0.68	0.70	2.60		0.79	0.65	0.70	0.77	3.00	
12	Leukemia/lymphoma	0.90	0.90	0.90	0.89	1.80		1.00	0.91	0.95	1.00	1.90	
13	Esophageal cancer	0.90	0.90	0.90	0.89	1.80		0.90	1.00	0.95	0.89	1.60	
14	Other injuries	1.00	0.73	0.84	1.00	1.90		1.00	0.89	0.94	1.00	1.60	
15	Other infectious diseases	0.38	0.60	0.46	0.33	0.90		0.75	0.86	0.80	0.73	1.20	
16	Others	0.94	0.47	0.61	0.94	6.30		0.83	0.88	0.85	0.82	3.00	
	Total				0.80		0.93				0.85		0.96

**Table 4:** The concordance between initial diagnosis before VA and MRR results.

**Initial diagnosis**	**MRR UCOD**																
	**Stroke**	**Other** **cancers**	**Ischaemic** **heart** **diseases**	**Lung** **cancer**	**Chronic** **respiratory** **diseases**	**Stomach** **cancer**	**Other** **non-communicable** **diseases**	**Colorectal** **cancer**	**Other** **cardiovascular** **diseases**	**Falls**	**Diabetes**	**Leukemia/** **lymphoma**	**Esophageal** **cancer**	**Other** **injuries**	**Other** **infectious** **diseases**	**Others**	**Sum**
Stroke	96	1	3		1				6		3						110
Other cancers		78		1			1						1				81
Ischaemic heart diseases	2		64		5		1	1	2								75
Lung cancer				67		1											68
Chronic respiratory diseases		1	1		53		1								1		57
Stomach cancer		1		1		27					1						30
Other non-communicable diseases	1						15		1	1							18
Colorectal cancer		1						18	1							1	21
Other cardiovascular diseases			1						5			1					7
Falls	1		1		1					13							16
Diabetes	1	1			1		1				10				1		15
Leukemia/lymphoma		1										9					10
Esophageal cancer		1											9				10
Other injuries	1				1					1				8			11
Other infectious diseases					1		1								3		5
Others		4	2		1		6		2	1					3	17	36
Sum	102	89	72	69	64	28	26	19	17	16	14	10	10	8	8	18	570

**Table 5:** The concordance between post-VA diagnosis and MRR results.

**Post-VA diagnosis**	**MRR UCOD**																
	**Stroke**	**Other** **cancers**	**Ischaemic** **heart** **diseases**	**Lung** **cancer**	**Chronic** **respiratory** **diseases**	**Stomach** **cancer**	**Other** **non-communicable** **diseases**	**Colorectal** **cancer**	**Other** **cardiovascular** **diseases**	**Falls**	**Diabetes**	**Leukemia** **/lymphoma**	**Esophageal** **cancer**	**Other** **injuries**	**Other** **infectious** **diseases**	**Others**	**Sum**
Stroke	98	1	2						7	1	2						111
Other cancers		84		2			1						1			2	90
Ischaemic heart diseases	2		67		3		1		2								75
Lung cancer				67													67
Chronic respiratory diseases					58		1		1						1		61
Stomach cancer		1				28					1						30
Other non-communicable diseases		1			1		21		2	2							27
Colorectal cancer								19	1								20
Other cardiovascular diseases			1		1				4								6
Falls	1		1							11							13
Diabetes	1	1			1		2				11				1		17
Leukemia/lymphoma		1										10					11
Esophageal cancer													9				9
Other injuries										1				8			9
Other infectious diseases															6	1	7
Others			1							1						15	17
Sum	102	89	72	69	64	28	26	19	17	16	14	10	10	8	8	18	570

Sensitivity and positive predictive value (PPV) were both high for the top six CODs. PPV was low for diabetes and other infectious diseases, indicating that some of the initial diagnoses that were not diabetes or other infectious diseases were reallocated to other diseases after the VA investigation.

Although not dramatic, overall CSMF accuracy improved from 0.93, based on the initial diagnoses, to 0.96 after the application of SmartVA (as shown in Table 3). In terms of specific causes, the CCCs for the top six causes of death (stroke, other cancers, ischemic heart disease (IHD), lung cancer, CRD, and stomach cancer, accounting for over 75% of deaths) all increased to more than 0.90 after VA-assisted diagnosis. Detailed metrics are shown in Tables 3–5. Some CODs, especially other non-communicable diseases (NCDs) and other infectious diseases, had noticeable increases in CCC following the application of SmartVA. Of interest is the change in CCC for other cardiovascular diseases (CVDs) and falls; both decreased after the VA investigation. This suggests that CVDs are being used as a convenient diagnostic category for some deaths, possibly those where it was difficult to establish the UCOD from the outpatient clinical records, which were subsequently reclassified following an investigation with SmartVA.

Table 4 shows the misclassification matrix based on the initial diagnosis compared with that from the MRR and is thus a rigorous test of the diagnostic accuracy of existing practices in the CHCs; 86.3% (492/570) of cases were correctly diagnosed by the initial diagnosis. The extent of misclassification was reduced following the VA investigation (Tables 4, 5), with overall diagnostic accuracy increasing to 90.5% (516/570) among the post-VA diagnoses.

Based on the results of the initial diagnosis before the VA investigation, other CVDs and other infectious diseases were more likely to be mis-assigned to other causes; nearly one-third of other CVDs were misclassified as stroke (6/17; Table 4).

The accuracy of CSMFs increased following the application of SmartVA, except for the categories of other CVDs and falls (Table 3). As mentioned, other CVDs were often (6/17 or 35.3%) misclassified cases of stroke, when compared with the MRR diagnoses (Table 5).

Analysis of the VA results with SmartVA Auto Analyze resulted in the causes of 53 deaths, or just under 10% of the sample, being reclassified from their initially assigned causes. This was particularly the case for chronic kidney diseases (CKDs), CRD, and cirrhosis, as well as falls, IHDs, other CVDs, and undetermined causes.

Among the 53 cases where the method led to a change in the COD, only 22.6% (12/53) were assigned correctly before VA (Table 6), whereas 67.9% (36/53) of the new CODs were assigned correctly according to MRR (Table 7). The number of misclassified conditions, compared with MRR, was also reduced. Among the 53 cases with a change in COD, all the causes assigned before VA (Table 6) had a high degree of misclassification, except for cirrhosis and falls. After VA, the misclassification was greatly reduced, except for falls and other CVDs (Table 7). Four undetermined deaths were reallocated to other diagnoses (as shown in Tables 3–8).

**Table 6:** The concordance between initial diagnosis and MRR results of the 53 changed cases where SmartVA led to a change in diagnosis.

**Initial diagnosis**	**MRR UCOD**															
	**Chronic** **respiratory** **diseases**	**Cirrhosis**	**Colorectal** **cancer**	**Diabetes**	**Falls**	**Ischaemic** **heart** **diseases**	**Leukemia/** **lymphoma**	**Lung** **cancer**	**Other** **cancers**	**Other** **cardiovascular** **diseases**	**Other** **infectious** **diseases**	**Other** **non-communicable** **diseases**	**Prostate** **cancer**	**Stomach** **cancer**	**Stroke**	**Total**
Cervical cancer									1							1
Chronic kidney disease						1						4				5
Chronic respiratory diseases	2					1			1			1				5
Cirrhosis		1							1		3					5
Colorectal cancer									1				1			2
Esophageal cancer									1							1
Falls	1				3											4
Ischaemic heart diseases	2		1			1										4
Lung cancer								1						1		2
Other cardiovascular diseases						1	1			2						4
Other infectious diseases	1											1				2
Other injuries	1				1										1	3
Other non-communicable diseases												1			1	2
Pneumonia	1									1						2
Prostate cancer									2				1			3
Stomach cancer								1								1
Stroke	1			1		1										3
Undetermined						1				1		2				4
Total	9	1	1	1	4	6	1	2	7	4	3	9	2	1	2	53

**Table 7:** The concordance between post-VA diagnosis and MRR results of the 53 changed cases where SmartVA led to a change in diagnosis.

**Post-VA diagnosis**	**MRR UCOD**															
	**Chronic respiratory diseases**	**Cirrhosis**	**Colorectal cancer**	**Diabetes**	**Falls**	**Ischaemic heart diseases**	**Leukemia/ lymphoma**	**Lung cancer**	**Other cancers**	**Other cardiovascular diseases**	**Other infectious diseases**	**Other non-communicable diseases**	**Prostate cancer**	**Stomach cancer**	**Stroke**	**Total**
Chronic respiratory diseases	7									1		1				9
Cirrhosis						1										1
Colorectal cancer			1													1
Diabetes				1								1				2
Falls					1											1
Ischaemic heart diseases						4										4
Leukemia/lymphoma							1									1
Lung cancer								1								1
Other cancers								1	6				2			9
Other cardiovascular diseases	1					1				1						3
Other infectious diseases		1									3					4
Other injuries					1											1
Other non-communicable diseases	1				1				1	1		7				11
Stomach cancer														1		1
Stroke					1					1					2	4
Total	9	1	1	1	4	6	1	2	7	4	3	9	2	1	2	53

**Table 8:** Validation metrics comparing initial diagnosis, any Tariff or post-VA diagnosis, with MRR UCOD (top 15 specific UCOD) of the 53 cases where diagnosis changed.

**Rank**	**UCOD**	**INI**						**Any tariff**						**Post-VA diagnosis**					
		**Sensitivity**	**PPV**	**Kappa**	**CCC**	**CSMF**	**CSMF accuracy**	**Sensitivity**	**PPV**	**Kappa**	**CCC**	**CSMF**	**CSMF accuracy**	**Sensitivity**	**PPV**	**Kappa**	**CCC**	**CSMF**	**CSMF accuracy**
1	Chronic respiratory diseases	0.29	0.40	0.21	0.23	13.5		1.00	0.82	0.87	1.00	28.2		0.78	0.78	0.73	0.76	17.3	
2	Cirrhosis	1.00	0.20	0.30	1.00	13.5		–	0.00	0.00	–	2.6		0.00	0.00	−0.02	−0.07	1.9	
3	Colorectal cancer	0.00	0.00	−0.04	−0.07	5.4		0.00	–	0.00	−0.07	0.0		1.00	1.00	1.00	1.00	1.9	
4	Diabetes	0.00	–	0.00	−0.07	0.0		1.00	0.50	0.65	1.00	5.1		1.00	0.50	0.66	1.00	3.8	
5	Falls	1.00	0.75	0.84	1.00	10.8		1.00	0.67	0.79	1.00	7.7		0.33	1.00	0.49	0.29	1.9	
6	Ischaemic heart diseases	0.25	0.25	0.16	0.20	10.8		0.67	0.50	0.48	0.64	20.5		0.67	1.00	0.78	0.64	7.7	
7	Leukemia/lymphoma	0.00	–	0.00	−0.07	0.0		0.00	–	0.00	−0.07	0.0		1.00	1.00	1.00	1.00	1.9	
8	Lung cancer	0.50	0.50	0.47	0.46	5.4		1.00	0.67	0.79	1.00	7.7		0.50	1.00	0.66	0.46	1.9	
9	Other cancers	0.00	–	0.00	−0.07	0.0		0.25	1.00	0.37	0.20	2.6		0.86	0.67	0.71	0.85	17.3	
10	Other cardiovascular diseases	1.00	0.50	0.64	1.00	10.8		0.00	–	0.00	−0.07	0.0		0.25	0.33	0.24	0.20	5.8	
11	Other infectious diseases	0.00	0.00	−0.07	−0.07	5.4		0.67	1.00	0.79	0.64	5.1		1.00	0.75	0.85	1.00	7.7	
12	Other non-communicable diseases	0.33	0.50	0.36	0.29	5.4		0.00	–	0.00	−0.07	0.0		0.78	0.64	0.63	0.76	21.2	
13	Prostate cancer	0.50	0.33	0.36	0.46	8.1		1.00	0.67	0.79	1.00	7.7		0.00	–	0.00	−0.07	0.0	
14	Stomach cancer	0.00	0.00	−0.03	−0.07	2.7		0.00	0.00	−0.04	−0.07	12.8		1.00	1.00	1.00	1.00	1.9	
15	Stroke	0.00	0.00	−0.04	−0.07	8.1		0.00	–	0.00	−0.07	0.0		1.00	0.50	0.65	1.00	7.7	
					0.28		0.69				–		0.67				0.59		0.84

For the 53 deaths where the UCOD changed after the application of SmartVA, the initially assigned CODs (initial diagnoses) were distributed reasonably randomly across the 15 causes. In the initially assigned CODs, no cases were assigned to leukemia/lymphoma, diabetes, or other cancers. However, according to the MRR results, other cancers should be the third leading COD in this sample of 53 deaths. SmartVA suggested that the fraction was 17%. CRD was only half as important as a cause (9.4 vs. 17%) according to the initial diagnosis compared with both SmartVA and the MRR. CKD, undetermined causes, other injuries, pneumonia, cervical cancer, and esophageal cancer were not among the UCOD identified by the MRR, or by SmartVA (except for other injuries), while the CHC doctors assigned them as UCODs after initial diagnosis. Overall, the CSMF pattern identified by the application of SmartVA for these 53 cases was much closer to the true pattern suggested from MRR than the initial diagnosis. This suggests a need for greater care when assigning these diseases as UCODs (Tables 6, 7).

With the assistance of SmartVA, the majority of misdiagnosed deaths were assigned to other NCDs (20.8%), CRD (17.0%), and other cancers (17.0%). Though a small degree of misclassification persisted, the post-VA diagnosis of the UCOD agreed more closely with the reference standard (MRR) than the initial diagnosis (Table 9).

**Table 9:** The distribution of UCOD for 53 misclassified cases (based on initial diagnosis).

	**Changed cause**	**Initial (%)**	**Post-VA (%)**	**MRR (%)**
1	Cervical cancer	1.9	0	0
2	Chronic kidney disease	9.4	0	0
3	Chronic respiratory diseases	9.4	17.0	17.0
4	Cirrhosis	9.4	1.9	1.9
5	Colorectal cancer	3.8	1.9	1.9
6	Esophageal cancer	1.9	0	0
7	Falls	7.5	1.9	7.5
8	Ischaemic heart diseases	7.5	7.5	11.3
9	Lung cancer	3.8	1.9	3.8
10	Other cardiovascular diseases	7.5	5.7	7.5
11	Other infectious diseases	3.8	7.5	5.7
12	Other injuries	5.7	1.9	0
13	Other non-communicable diseases	3.8	20.8	17.0
14	Pneumonia	3.8	0	0
15	Prostate cancer	5.7	0	3.8
16	Stomach cancer	1.9	1.9	1.9
17	Stroke	5.7	7.5	3.8
18	Undetermined	7.5	0	0
19	Diabetes	0	3.8	0
20	Leukemia/lymphoma	0	1.9	1.9
21	Other cancers	0	17.0	13.2

## Discussion

Although Shanghai has an established and well-functioning CRVS system, SmartVA for Physicians contributed to an improvement in the accuracy of death certification, as measured by the CSMF, which increased from 0.93 to 0.96 following the introduction of SmartVA. In addition, SmartVA may be a useful tool for inferring some special causes of death, such as those CODs classified as undetermined, which while less of an issue for Shanghai, is a common problem in civil registration systems worldwide (20–24). In our study, four undetermined CODs were reclassified after the application of SmartVA. With the help of this tool, the Shanghai CRVS system could reduce the fraction of undetermined deaths.

Among the 53 cases where the UCOD was misclassified according to the VA investigation, the largest impact was for CRD (17 vs. 9.4% suggested by initial diagnosis), other NCDs (17 vs. 3.8%), as well as other cancers (13.2 vs. 0%), suggesting that for causes such as these, a more careful examination of the available medical history may be needed by the certifier before assigning the UCOD. The fact that only 53 cases were misclassified out of a sample of 570 reflects the rigor of the diagnostic practices routinely applied in Shanghai, but given the clustering of these cases around certain causes of death (COPD, residual NCDs, and residual cancers), selective application of the methodology might help to improve diagnostic accuracy even further.

The improvement in COD data following the application of SmartVA in this study could be attributed to several factors. First, the SOPs for COD assignment that were followed during the SmartVA investigation ensured a structured and consistent approach, leading to a more accurate COD assignment. Second, the SmartVA procedure has systematic and comprehensive questions about symptoms, which can help to ensure that all relevant medical information regarding the decedent's morbid conditions is captured at the time of certification of the COD. Third, the improvement attributed to Smart VA could in part be due to the comprehensive training in seeking information about symptoms and signs from the family, which is more systematic and comprehensive than current procedures.

As Shanghai is highly developed with a relatively advanced CRVS system, the routine use of SmartVA is unlikely to result in a significant improvement in the accuracy of COD data in the Shanghai system, nor is it a cost-effective way to do so. The routine application of SmartVA would add a further 15–30 min to the diagnostic process for each death, which, given the already high diagnostic standards and procedures in place, is not justifiable. Shanghai CHC doctors' routine work already comprises checking and correcting MCCOD data, including re-interviewing the family of the deceased. In contrast, for other cities in China, especially in the remote areas in the west, that do not have a well-functioning death registration system, SmartVA may be more beneficial.

There are several reasons why not all the home deaths can be investigated. The high refusal rate undoubtedly reflects the fact that urban, comparatively well-off populations engaged in non-agricultural occupations have little time or inclination to respond to questionnaires, particularly at a time when the family of the deceased is still grieving, making the investigation more difficult to conduct. Second, conducting the SmartVA investigation requires systematic training from an expert team. Aside from regular medical certification of COD training, the training courses include how to install the software for the SmartVA tool, how to connect the tablet to the computer to transmit the survey data, etc. This is further complicated by the mobility of CHC physicians, which is quite high as their workload is heavy. Shanghai CDC has subsequently developed the WeChat version of the Smart VA questionnaire in 2021 and is conducting a new round of home death investigation for those UCODs which were initially assigned as R codes.

Our SmartVA study has some limitations. First, the SmartVA tool, especially the cause list, is not perfectly suited to the actual mortality fractions observed in Shanghai (25). For example, liver cancer is not on the SmartVA cause list, and therefore the program does not assign it as a COD to any deaths, whereas liver cancer accounted for more than 2.6% of all deaths in Shanghai in 2018. This is due to the fact that validation metrics for liver cancer in the original PHMRC study were considered too low to justify the inclusion of liver cancer as a target cause for SmartVA (11, 12). Second, in this study, the VA investigations were conducted after the certifier reviewed the previous outpatient medical histories of the deceased. This may have biased the certifier when considering the diagnostic information suggested by SmartVA. Third, in this study in Shanghai, the SmartVA procedure was implemented in 16 communities. As the number of deaths in these communities was not very high and may not be representative of the whole population, further research should be done to determine the generalizability of SmartVA for Physicians before it can be extended to all districts and counties in China where home deaths are common. Fourth, the GS dataset used in the MRR to establish true causes of death is not without errors, given that it has been derived from available medical records, which can themselves contain errors. The surest way to ensure an error free GS is through autopsy, but this is not practical or affordable in most settings. Rather, by adopting *ex-ante* diagnostic procedures with clearly defined diagnostic criteria for specific causes of interest, the PHMRC methods and exclusion criteria applied in this study are likely to dramatically reduce, but not eliminate, diagnostic uncertainty and subjectivity. Last, the cause pattern identified by SmartVA is constrained to the causes associated with the symptom questions asked in SmartVA. While these causes collectively would likely account for the vast majority of deaths in most low- or middle-income countries, important local causes, such as liver cancer in China, may be omitted due to the criteria and methods used to validate the tool.

While we have focused on the applicability of SmartVA in the Shanghai context, it should be kept in mind that there are alternative automated (electronic) VA methods, such as *In Silico* VA and InterVA, which could also be applied to assist physician diagnoses (26–28). In our study, the strengths and weaknesses of different automated diagnostic methods were not discussed. Rather, we have focused on the applicability of generic automated VA methods as a diagnostic aid for physicians who need to certify the cause of home deaths, often in the absence of good clinical records (29–33).

The SCDC plans to adapt the workflow and operational specifications of SmartVA to maximize the effectiveness of the method in improving COD diagnosis and lowering the proportion of undetermined causes of death. This will most likely be through selective application and integration into the existing CRVS system. In addition, SCDC is also considering using SmartVA to identify the possible causes of death in cases with incomplete medical history information.

Increasing the diagnostic accuracy of any dataset that is likely to be used to guide public policy is, or should be, a priority for data custodians. Our research has demonstrated that COD accuracy in Shanghai is very good, but it is not without errors. Furthermore, our study has shown that the application of SmartVA can improve diagnostic accuracy even further, if only marginally. As a result, the routine use of SmartVA is unlikely to be a cost-effective strategy to further improve the diagnostic accuracy of an already well-performing system, but its application to improve the diagnoses of certain conditions appears justified. This marginal application would likely further improve confidence in the use of Shanghai COD data for some public health purposes.

## Conclusion

This research illustrates that although Shanghai has an established and well-functioning CRVS system, SmartVA for Physicians contributed to an improvement in the accuracy of death certification. In addition, SmartVA may be a useful tool for inferring some special causes of death, such as those CODs classified as undetermined.

## Data Availability Statement

The data that support the findings of this study are available upon request from the Shanghai Municipal Center for Disease Control and Prevention (Shanghai CDC) but restrictions apply to the availability of these data, which were used under license for the current study, and so are not publicly available. Data are available from the authors upon reasonable request and with the permission of Shanghai CDC.

## Ethics Statement

The studies involving human participants were reviewed and approved by Ethics approval was obtained from Shanghai CDC (Ethics ID: 2016-28) and the University of Melbourne Ethics Committees (Ethics ID: 1647517.1.1). The patients/participants provided their written informed consent to participate in this study.

## Author Contributions

RJ and AL devised the study and were responsible for the study design. ZY, TX, and CW oversaw the research. LC and HL were members of the writing group. HL, TA, CW, HY, and AL provided feedback on data analysis, results, and discussion. RR, ZG, BF, AL, and DM revised the manuscript critically for important intellectual content. All authors contributed to the framework construction, results interpretation, manuscript revision, and approved the final version of the manuscript. The corresponding authors attest that all listed authors meet authorship criteria and that no others meeting the criteria have been omitted.

## Funding

The research was funded by the Bloomberg Philanthropies Data for Health Initiative and by the Clinical Research Project of the Health Industry of Shanghai Health Commission in 2020 (Award number: 20204Y0205). The funders had no role in study design, data collection and analysis, decision to publish, or the preparation of the manuscript.

## Conflict of Interest

The authors declare that the research was conducted in the absence of any commercial or financial relationships that could be construed as a potential conflict of interest.

## Publisher's Note

All claims expressed in this article are solely those of the authors and do not necessarily represent those of their affiliated organizations, or those of the publisher, the editors and the reviewers. Any product that may be evaluated in this article, or claim that may be made by its manufacturer, is not guaranteed or endorsed by the publisher.

## Abbreviations

CHC: community health centers
VA: verbal autopsy
COD: cause of death
PHMRC: Population Health Metrics Research Consortium
ODK: Open Data Kit
SOPs: Standard Operating Procedures
SCDC: Shanghai Municipal Center for Disease Control and Prevention
UCOD: underlying cause of death
GS: gold standard
MRR: medical record review
CSMF: cause-specific mortality fraction
CCC: chance-corrected concordance
CKD: chronic kidney diseases
IHD: ischemic heart diseases
CVD: cardiovascular diseases
CRD: chronic respiratory diseases.
