# Supplementary material for: Assessing the Diagnostic Accuracy of Physicians for Home Death Certification in Shanghai: Application of SmartVA
Source: Front Public Health. 2022 Jun 17;10:842880. doi: 10.3389/fpubh.2022.842880 (PMC9247331; doi:10.3389/fpubh.2022.842880)
Supplement: Supplementary file 1 [file Data_Sheet_1.docx]

**Supplementary table 1 The concordance between Tariff1 and MRR results of the 53 changed cases**

| Tariff1 | MRR UCOD | | | | | | | | | | | | | | | |
| --- | --- | --- | --- | --- | --- | --- | --- | --- | --- | --- | --- | --- | --- | --- | --- | --- |
|  | Chronic Respiratory diseases | Cirrhosis | Colorectal Cancer | Diabetes | Falls | Ischaemic Heart Diseases | Leukemia/ Lymphoma | Lung Cancer | Other Cancers | Other Cardiovascular Diseases | Other Infectious Diseases | Other Non-communicable Diseases | Prostate Cancer | Stomach Cancer | Stroke | Sum |
| Cervical Cancer |  |  |  | 1 |  |  |  |  | 1 |  |  |  |  |  |  | 2 |
| Chronic Kidney Disease |  |  |  |  |  |  |  |  |  |  |  | 3 |  |  |  | 3 |
| Chronic Respiratory diseases | 2 |  |  |  |  | 1 |  |  | 1 |  |  |  | 1 |  |  | 5 |
| Cirrhosis |  |  |  |  |  |  |  |  | 1 |  | 1 |  |  |  |  | 2 |
| Diabetes |  |  |  |  |  |  |  |  |  |  |  | 1 |  |  |  | 1 |
| Diarrhea/Dysentery |  |  |  |  |  |  |  |  | 1 |  |  |  |  |  |  | 1 |
| Esophageal Cancer |  |  |  |  |  |  |  |  | 1 |  |  |  |  |  |  | 1 |
| Falls |  |  |  |  | 2 |  |  |  |  |  |  |  |  |  | 1 | 3 |
| Ischaemic Heart Diseases | 3 |  | 1 |  |  | 4 | 1 | 1 |  |  | 1 | 1 |  |  |  | 12 |
| Lung Cancer |  |  |  |  |  |  |  | 1 |  |  |  |  | 1 | 1 |  | 3 |
| Other Infectious Diseases |  |  |  |  |  |  |  |  |  |  | 1 |  |  |  |  | 1 |
| Other Injuries |  |  |  |  | 2 |  |  |  |  |  |  |  |  |  |  | 2 |
| Pneumonia | 1 |  |  |  |  |  |  |  |  |  |  | 1 |  |  |  | 2 |
| Prostate Cancer |  |  |  |  |  |  |  |  | 2 |  |  |  |  |  |  | 2 |
| Stroke | 2 |  |  |  |  | 1 |  |  |  | 2 |  | 1 |  |  | 1 | 7 |
| TB | 1 |  |  |  |  |  |  |  |  |  |  |  |  |  |  | 1 |
| Undetermined |  | 1 |  |  |  |  |  |  |  | 2 |  | 2 |  |  |  | 5 |
| Sum | 9 | 1 | 1 | 1 | 4 | 6 | 1 | 2 | 7 | 4 | 3 | 9 | 2 | 1 | 2 | 53 |

**Supplementary table 2 The concordance between Tariff2 and MRR results of the 53 changed cases**

| Tariff 2 | MRR UCOD | | | | | | | | | | | | | | | | |
| --- | --- | --- | --- | --- | --- | --- | --- | --- | --- | --- | --- | --- | --- | --- | --- | --- | --- |
|  | Chronic Respiratory diseases | Cirrhosis | Colorectal Cancer | Diabetes | Falls | Ischaemic Heart Diseases | Leukemia/ Lymphoma | Lung Cancer | Other Cancers | Other Cardiovascular Diseases | Other Infectious Diseases | Other Non-communicable Diseases | Prostate Cancer | Stomach Cancer | Stroke | Sum |  |
| Chronic Kidney Disease |  |  |  |  |  | 3 |  |  |  |  |  | 1 |  |  |  | 4 |  |
| Chronic Respiratory diseases | 6 |  | 1 |  |  | 1 |  |  |  |  |  | 1 |  | 1 |  | 10 |  |
| Cirrhosis |  |  |  |  |  |  |  |  | 1 |  |  |  |  |  |  | 1 |  |
| Diabetes |  |  |  | 1 |  |  |  |  |  |  | 1 | 2 |  |  |  | 4 |  |
| Esophageal Cancer |  |  |  |  |  |  |  |  | 1 |  |  |  |  |  |  | 1 |  |
| Ischaemic Heart Diseases | 1 |  |  |  |  |  |  |  |  |  |  | 1 |  |  |  | 2 |  |
| Lung Cancer |  |  |  |  |  |  |  | 1 | 1 |  |  |  |  |  |  | 2 |  |
| Other Infectious Diseases |  |  |  |  |  |  | 1 |  | 1 |  | 1 |  |  |  |  | 2 |  |
| Other Non-communicable Diseases |  |  |  |  |  |  |  |  | 1 |  |  |  |  |  |  | 1 |  |
| Pneumonia | 2 |  |  |  |  |  |  |  |  | 1 |  |  |  |  |  | 3 |  |
| Prostate Cancer |  |  |  |  |  |  |  | 1 | 2 |  |  |  | 2 |  |  | 5 |  |
| NA |  | 1 |  |  | 4 | 2 | 1 |  |  | 3 | 1 | 4 |  |  | 2 | 18 |  |
| Sum | 9 | 1 | 1 | 1 | 4 | 6 | 1 | 2 | 7 | 4 | 3 | 9 | 2 | 1 | 2 | 53 |  |

**Supplementary table 3 The concordance between Tariff3 and MRR results of the 53 changed cases**

| Tariff 3 | MRR UCOD | | | | | | | | | | | | | | | |
| --- | --- | --- | --- | --- | --- | --- | --- | --- | --- | --- | --- | --- | --- | --- | --- | --- |
|  | Chronic Respiratory diseases | Cirrhosis | Colorectal Cancer | Diabetes | Falls | Ischaemic Heart Diseases | Leukemia/ Lymphoma | Lung Cancer | Other Cancers | Other Cardiovascular Diseases | Other Infectious Diseases | Other Non-communicable Diseases | Prostate Cancer | Stomach Cancer | Stroke | Sum |
| Chronic Respiratory diseases | 1 |  |  |  |  |  |  |  |  |  |  |  |  |  |  | 1 |
| Cirrhosis |  |  |  |  |  |  |  |  |  |  | 1 |  |  |  |  | 1 |
| Ischaemic Heart Diseases | 1 |  |  |  |  |  |  |  |  | 1 |  |  |  |  |  | 2 |
| Lung Cancer |  |  |  |  |  |  |  |  | 1 |  |  |  | 1 |  |  | 2 |
| Other Cancers |  |  |  |  |  |  |  |  | 1 |  |  |  |  |  |  | 1 |
| Other Non-communicable Diseases | 1 |  |  |  |  | 1 |  |  | 2 |  |  |  |  |  |  | 4 |
| Pneumonia | 2 |  |  |  |  |  |  |  |  |  |  |  |  |  |  | 2 |
| Prostate Cancer |  |  |  |  |  |  |  | 1 | 1 |  |  |  |  | 1 |  | 3 |
| Stomach Cancer |  |  |  |  |  |  |  |  | 1 |  |  |  |  |  |  | 1 |
| Stroke | 1 |  | 1 | 1 |  |  |  |  |  |  |  |  |  |  |  | 3 |
| TB | 1 |  |  |  |  |  |  |  |  |  |  |  |  |  |  | 1 |
| NA | 2 | 1 |  |  | 4 | 5 | 1 | 1 | 1 | 3 | 2 | 9 | 1 |  | 2 | 32 |
| Sum | 9 | 1 | 1 | 1 | 4 | 6 | 1 | 2 | 7 | 4 | 3 | 9 | 2 | 1 | 2 | 53 |

**Supplementary table 4 The concordance between any Tariff that equals MRR UCOD versus MRR UCOD of the changed 53 cases**

| any Tariff that equals MRR UCOD | MRR UCOD | | | | | | | | | | | | | | | |
| --- | --- | --- | --- | --- | --- | --- | --- | --- | --- | --- | --- | --- | --- | --- | --- | --- |
|  | Chronic Respiratory diseases | Cirrhosis | Colorectal Cancer | Diabetes | Falls | Ishaemic Heart Diseases | Leukemia/ Lymphoma | Lung Cancer | Other Cancers | Other Cardiovascular Diseases | Other Infectious Diseases | Other Non-communicable Diseases | Prostate Cancer | Stomach Cancer | Stroke | Sum |
| Cervical Cancer |  |  |  |  |  |  |  |  | 1 |  |  |  |  |  |  | 1 |
| Chronic Kidney Disease |  |  |  |  |  |  |  |  |  |  |  | 3 |  |  |  | 3 |
| Chronic Respiratory diseases | 9 |  |  |  |  | 1 |  |  | 1 |  |  |  |  |  |  | 11 |
| Cirrhosis |  |  |  |  |  |  |  |  | 1 |  |  |  |  |  |  | 1 |
| Diabetes |  |  |  | 1 |  |  |  |  |  |  |  | 1 |  |  |  | 2 |
| Diarrhea/Dysentery |  |  |  |  |  |  |  |  | 1 |  |  |  |  |  |  | 1 |
| Esophageal Cancer |  |  |  |  |  |  |  |  | 1 |  |  |  |  |  |  | 1 |
| Falls |  |  |  |  | 2 |  |  |  |  |  |  |  |  |  | 1 | 3 |
| Ishaemic Heart Diseases |  |  | 1 |  |  | 4 | 1 |  |  |  | 1 | 1 |  |  |  | 8 |
| Lung Cancer |  |  |  |  |  |  |  | 2 |  |  |  |  |  | 1 |  | 3 |
| Other Cancers |  |  |  |  |  |  |  |  | 1 |  |  |  |  |  |  | 1 |
| Other Infectious Diseases |  |  |  |  |  |  |  |  |  |  | 2 |  |  |  |  | 2 |
| Other Injuries |  |  |  |  | 2 |  |  |  |  |  |  |  |  |  |  | 2 |
| Pneumonia |  |  |  |  |  |  |  |  |  |  |  | 1 |  |  |  | 1 |
| Prostate Cancer |  |  |  |  |  |  |  |  | 1 |  |  |  | 2 |  |  | 3 |
| Stroke |  |  |  |  |  | 1 |  |  |  | 2 |  | 1 |  |  | 1 | 5 |
| Undetermined |  | 1 |  |  |  |  |  |  |  | 2 |  | 2 |  |  |  | 5 |
| Sum | 9 | 1 | 1 | 1 | 4 | 6 | 1 | 2 | 7 | 4 | 3 | 9 | 2 | 1 | 2 | 53 |

**Supplementary table 5 The metrics of the comparison between VA Tariff 1 and MRR after referring to the decision lists for the changed causes**

| Rank | UCOD | Tariff1 | | | | |  |
| --- | --- | --- | --- | --- | --- | --- | --- |
|  |  | Sensitivity | PPV | Kappa | CCC | CSMF | CSMF accuracy |
| 1 | Chronic Respiratory diseases | 0.29 | 0.40 | 0.20 | 0.23 | 13.90 |  |
| 2 | Cirrhosis | - | 0.00 | 0.00 | - | 5.60 |  |
| 3 | Colorectal Cancer | 0.00 | - | 0.00 | -0.07 | 0.00 |  |
| 4 | Diabetes | - | 0.00 | 0.00 | - | 2.80 |  |
| 5 | Falls | 1.00 | 0.67 | 0.79 | 1.00 | 8.30 |  |
| 6 | Ischaemic Heart Diseases | 0.67 | 0.33 | 0.29 | 0.64 | 33.30 |  |
| 7 | Leukemia/ Lymphoma | 0.00 | - | 0.00 | -0.07 | 0.00 |  |
| 8 | Lung Cancer | 0.50 | 0.33 | 0.36 | 0.46 | 8.30 |  |
| 9 | Other Cancers | 0.00 | - | 0.00 | -0.07 | 0.00 |  |
| 10 | Other Cardiovascular Diseases | 0.00 | - | 0.00 | -0.07 | 0.00 |  |
| 11 | Other Infectious Diseases | 0.33 | 1.00 | 0.48 | 0.29 | 2.80 |  |
| 12 | Other Non-communicable Diseases | 0.00 | - | 0.00 | -0.07 | 0.00 |  |
| 13 | Prostate Cancer | 0.00 | 0.00 | -0.06 | -0.07 | 5.60 |  |
| 14 | Stomach Cancer | 0.00 | - | 0.00 | -0.07 | 0.00 |  |
| 15 | Stroke | 0.50 | 0.14 | 0.15 | 0.46 | 19.40 |  |
|  |  |  |  |  | - |  | **0.56** |

**Supplementary table 6 The metrics of the comparison between VA Tariff 2 and MRR after referring to the decision lists for the changed causes**

| Rank | UCOD | Tariff2 | | | | |  |
| --- | --- | --- | --- | --- | --- | --- | --- |
|  |  | Sensitivity | PPV | Kappa | CCC | CSMF | CSMF accuracy |
| 1 | Chronic Respiratory diseases | 0.86 | 0.60 | 0.58 | 0.85 | 37.00 |  |
| 2 | Cirrhosis | - | 0.00 | 0.00 | - | 3.70 |  |
| 3 | Colorectal Cancer | 0.00 | - | 0.00 | -0.07 | 0.00 |  |
| 4 | Diabetes | 1.00 | 0.25 | 0.36 | 1.00 | 14.80 |  |
| 5 | Falls | - | - | - | - | 0.00 |  |
| 6 | Ischaemic Heart Diseases | 0.00 | 0.00 | -0.05 | -0.07 | 7.40 |  |
| 7 | Leukemia/ Lymphoma | - | - | - | - | 0.00 |  |
| 8 | Lung Cancer | 0.50 | 0.50 | 0.46 | 0.46 | 7.40 |  |
| 9 | Other Cancers | 0.00 | - | 0.00 | -0.07 | 0.00 |  |
| 10 | Other Cardiovascular Diseases | - | - | - | - | 0.00 |  |
| 11 | Other Infectious Diseases | 0.50 | 0.50 | 0.46 | 0.46 | 7.40 |  |
| 12 | Other Non-communicable Diseases | 0.00 | 0.00 | -0.06 | -0.07 | 3.70 |  |
| 13 | Prostate Cancer | 1.00 | 0.40 | 0.52 | 1.00 | 18.50 |  |
| 14 | Stomach Cancer | 0.00 | - | 0.00 | -0.07 | 0.00 |  |
| 15 | Stroke | - | - | - | - |  |  |
|  |  |  |  |  | - |  | **0.59** |

**Supplementary table 7 The metrics of the comparison between VA Tariff 3 and MRR after referring to the decision lists for the changed causes**

| Rank | UCOD | Tariff3 | | | | |  |
| --- | --- | --- | --- | --- | --- | --- | --- |
|  |  | Sensitivity | PPV | Kappa | CCC | CSMF | CSMF accuracy |
| 1 | Chronic Respiratory diseases | 0.25 | 1.00 | 0.34 | 0.20 | 5.60 |  |
| 2 | Cirrhosis | - | 0.00 | 0.00 | - | 5.60 |  |
| 3 | Colorectal Cancer | 0.00 | - | 0.00 | -0.07 | 0.00 |  |
| 4 | Diabetes | 0.00 | - | 0.00 | -0.07 | 0.00 |  |
| 5 | Falls | - | - | - | - | 0.00 |  |
| 6 | Ischaemic Heart Diseases | 0.00 | 0.00 | -0.08 | -0.07 | 11.10 |  |
| 7 | Leukemia/ Lymphoma | - | - | - | - | 0.00 |  |
| 8 | Lung Cancer | 0.00 | 0.00 | -0.08 | -0.07 | 11.10 |  |
| 9 | Other Cancers | 0.17 | 1.00 | 0.21 | 0.11 | 5.60 |  |
| 10 | Other Cardiovascular Diseases | 0.00 | - | 0.00 | -0.07 | 0.00 |  |
| 11 | Other Infectious Diseases | 0.00 | - | 0.00 | -0.07 | 0.00 |  |
| 12 | Other Non-communicable Diseases | - | 0.00 | 0.00 | - | 22.20 |  |
| 13 | Prostate Cancer | 0.00 | 0.00 | -0.09 | -0.07 | 16.70 |  |
| 14 | Stomach Cancer | 0.00 | 0.00 | -0.06 | -0.07 | 5.60 |  |
| 15 | Stroke | - | 0.00 | 0.00 | - | 16.70 |  |
|  |  |  |  |  | - |  | **0.33** |
