# Supplementary material for: Assessing the Diagnostic Accuracy of Physicians for Home Death Certification in Shanghai: Application of SmartVA
Source: Front Public Health. 2022 Jun 17;10:842880. doi: 10.3389/fpubh.2022.842880 (PMC9247331; doi:10.3389/fpubh.2022.842880)
Supplement: Supplementary file 2 [file Table_1.DOCX]

Supplementary file 1. Cause of death list for Smart Verbal Autopsy with Corresponding ICD-10 codes

| Text for Smart VA cause (Adult) | ICD-10 Codes (from ICD-10 ) |
| --- | --- |
| Diarrhea/Dysentery | A00-A09 |
| TB | A15-A19 |
| AIDS | B20-B24 |
| Malaria | B50-B54 |
| Other Infectious Diseases | A10-A14, A20-B19, B25-B49, B55-B99 |
| Esophageal Cancer | C15 |
| Stomach Cancer | C16 |
| Colorectal Cancer | C18-C21 |
| Lung Cancer | C34 |
| Breast Cancer | C50 |
| Cervical Cancer | C53 |
| Prostate Cancer | C61 |
| Leukemia/Lymphoma | C81-C85，C91-C96 |
| Other Cancers | C00-C14, C17, C22-C33, C35-C49, C51-C52, C54-C60, C62-C80, C86-C90, C97-D48 |
| Diabetes | E10-E14 |
| Other Cardiovascular  Diseases | I00-I19, I26-I59, I70-I99 |
| Ishaemic Heart Diseases | I20-I25 |
| Stroke | I60-I69 |
| Pneumonia | J10-J22, J85 |
| Chronic Respiratory diseases | J40-J46 |
| Cirrhosis | K70-K76 |
| Chronic Kidney Disease | N17-N19 |
| Maternal | O00-O99 |
| Undetermined | R00-R99 |
| Road Traffic | V01-V89 |
| Falls | W00-W19 |
| Drowning | W65-W74 |
| Fires | X00-X19 |
| Bite of Venomous Animal | X20-X29 |
| Poisonings (accidental) | X40-X49 |
| Suicide (intentional self- harm) | X60-X84 |
| Homicide (assault) | X85-Y09 |
| Other Injuries | S00-T98, V90-V99, W20-W64, W75-W99, X30-X39, X50-X59, Y10-Y98 |
| Other Non-communicable  Diseases | All other ICD-10 codes NCDs* |
